# Implementation of Microfluidics for Antimicrobial Susceptibility Assays: Issues and Optimization Requirements

**DOI:** 10.3389/fcimb.2020.547177

**Published:** 2020-09-17

**Authors:** Nicole C. Parsley, Amanda L. Smythers, Leslie M. Hicks

**Affiliations:** Department of Chemistry, University of North Carolina at Chapel Hill, Chapel Hill, NC, United States

**Keywords:** antimicrobial susceptibility testing, microfluidics, antimicrobial peptides, viola inconspicua, cyclotides, bioassay, 96-well plates, antibiotics

## Abstract

Despite the continuous emergence of multi-drug resistant pathogens, the number of new antimicrobials reaching the market is critically low. Natural product peptides are a rich source of bioactive compounds, and advances in mass spectrometry have achieved unprecedented capabilities for the discovery and characterization of novel molecular species. However, traditional bioactivity assay formats hinder the discovery and biochemical characterization of natural product antimicrobial peptides (AMPs), necessitating large sample quantities and significant optimization of experimental parameters to achieve accurate/consistent activity measurements. Microfluidic devices offer a promising alternative to bulk assay systems. Herein, a microfluidics-based bioassay was compared to the traditional 96-well plate format in respective commercially-available hardware. Bioactivity in each assay type was compared using a *Viola inconspicua* peptide library screened against *E. coli* ATCC 25922. Brightfield microcopy was used to determine bioactivity in microfluidic channels while both common optical and fluorescence-based measurements of cell viability were critically assessed in plate-based assays. Exhibiting some variation in optical density and fluorescence-based measurements, all plate-based assays conferred bioactivity in late eluting *V. inconspicua* library fractions. However, significant differences in the bioactivity profiles of plate-based and microfluidic assays were found, and may be derived from the materials comprising each assay device or the growth/assay conditions utilized in each format. While new technologies are necessary to overcome the limitations of traditional bioactivity assays, we demonstrate that off-the-shelf implementation of microfluidic devices is non-trivial and significant method development/optimization is required before conventional use can be realized for sensitive and rapid detection of AMPs in natural product matrices.

## Introduction

Major advances in twentieth century antimicrobial therapies significantly decreased mortality associated with microbial infections; however, a recent global surge of antimicrobial resistance (AMR) has made the treatment of common bacterial and fungal infections increasingly challenging. Once viable antimicrobial therapies are now obsolete as new mechanisms for AMR develop, accelerated by the “misuse and overuse” of antimicrobial drugs (World Health Organization, 2018). Additionally, most recent commercial drug discovery efforts have targeted chronic ailments, ushering in a decline in antimicrobial approvals over the past three decades (Cole, 2014). As such, the future of global health relies on an infusion of new antimicrobial therapeutics in the drug discovery pipeline.

Natural product bioactive peptides are a vast and largely untapped source of antimicrobial chemistries expressed by all life, offering innate antimicrobial, antiviral, and anticancer properties (Chen and Lu, 2020). Natural product drug discovery has often favored the identification and isolation of small molecule constituents; however, antimicrobial peptides (AMPs) offer a complementary activity profile with the potential for increased selectivity and efficacy against pathogens (Fosgerau and Hoffmann, 2015; Lázár et al., 2018). Peptide-based therapeutics debuted in the clinic in WWII with the soil bacilli-derived tyrothricin (a mixture of gramicidins and tyrocidines), a potent topical antimicrobial agent that predated the commercialization of penicillin (Wenzel et al., 2018). Despite the characterization of >3,000 AMPs since then (Wang et al., 2016), only seven peptide antimicrobial drugs are currently approved by the FDA (Divyashree et al., 2020). Antimicrobial peptide discovery, characterization, and ultimately the path to clinical relevance is hindered by extreme diversity in sequence, size (2 to >50 residues), charge, post-translational modification (e.g., cyclic, disulfide-bound), and natural product complexity.

To address this gap, PepSAVI-MS was developed to identify low abundance bioactive peptides in complex natural product extracts (Kirkpatrick et al., 2017, 2018a,b; Moyer et al., 2019; Parsley et al., 2019). PepSAVI-MS is a top-down peptidomics approach that leverages modern mass spectrometry and relies on traditional bioassays for bioactivity characterization (e.g., 96-well plate or disk-diffusion). The sample quantities needed (~ micrograms) are often a limiting factor toward the rapid identification of novel AMPs. Plate-based assays offer increased sensitivity and higher throughput compared with traditional agar disk-diffusion assays, and are largely accessible in inexpensive, commercially-available 6, 12, 24, 96, and 384-well formats. Although higher density wells allow for greater throughput and require less sample volume per well, wells with smaller volumes (e.g., 384-well) can inhibit pathogen growth due to challenges with oxygenation (Duetz and Witholt, 2004) and mixing. (Walling et al., 2007; Regnault et al., 2018). Plate-based assays require optimization regarding pathogen identity, cell seeding density (Che et al., 2009), aeration (Duetz and Witholt, 2004), media choice (Gomez-Lopez et al., 2005; Agarwal et al., 2011), and assay length (Agarwal et al., 2011), and are unable to replicate physiological conditions.

Optical density (O.D) (Stevenson et al., 2016) and fluorescence-based measurements (Riss et al., 2004; Osaka and Hefty, 2013) are among the most commonly used methods to determine cell viability in plate-based assays. Optical density reads are often hindered by a low dynamic range and are highly susceptible to inhomogeneities of the solution tested, which can be problematic with bacterial cells prone to clumping and biofilm formation (Stevenson et al., 2016). Frequently used as a measurement directly proportional to cell density, O.D. must be calibrated based on specific species and measurement conditions (Stevenson et al., 2016). Metabolically-driven fluorescent dyes, e.g., resazurin/resorufin, have a large dynamic range, however; these are subject to extensive non-specific interactions with non-bacterial samples components (particularly thiol and carboxylic acid moieties)—a major cause of false positives/negatives (Neufeld et al., 2018). Resazurin must be intracellularly aerobically reduced by metabolically active cells to fluorescent resorufin, requiring >30 min incubation time prior to analysis (Chen et al., 2018; Kim and Jang, 2018), and the ability of bacterial species to participate in this reduction can be strain dependent due to differences in membrane permeability. While resazurin is effectively used for endpoint analysis, the further reduction of resorufin into a colorless non-fluorescent product makes it challenging to monitor cell growth over time (O'Brien et al., 2000). Despite common use in testing for antibiotic susceptibility, both O.D. and fluorescence-based bioactivity assays often require significant optimization prior to reliable use (Stevenson et al., 2016; Uzarski et al., 2017; Kim and Jang, 2018). Importantly, plate-based assays offer only a macroscopic view of indirectly measured bioactivity and provide no insight into cellular effects of treatment. Alternative assay formats with minimized optimization requirements and sample consumption are needed to enable rapid and accurate bioactivity assessment.

Emerging and highly versatile microfluidic technologies offer an alternative format for bioactivity testing; operating at micrometer scales and down to picoliter volumes, microfluidic platforms offer significant experimental flexibility via customizable fabrications, where channels and chambers may be precisely designed to answer specific biological questions. These technologies enable bioactivity assessment through microscopic detection of bacterial growth/inhibition, facilitating the direct evaluation of AMP mechanism of action, and boast minute sample/reagent requirements, decreased assay times, and highly controlled environmental conditions (Liu et al., 2017). Microfluidics have been used to assay “unculturable” bacteria while screening for bioactive compounds with higher sensitivity and in a 4-24x decreased time frame needed for traditional approaches (Behera et al., 2019). Furthermore, microfluidic platforms may better imitate physiological microsystems (e.g., blood vessels and anoxygenic environments), supporting clinically-relevant quantitative evaluation of AMP efficacy that supersedes that of bulk assays (Kim et al., 2010).

Microfluidic platforms are adaptable, allowing for cell viability analysis via fluorescent/cell staining dyes and through direct cell counting with the acquisition of brightfield images (Duncombe et al., 2015). Metabolically/biomolecule-specific fluorescent and cell staining dyes (e.g., Hoechst 33342) can be incorporated into microfluidic platforms to provide insight into mechanism of action and the differential effects of antibiotic treatment on inhomogeneous cell populations. Although the use of fluorescence-based assays must often contend with cellular phototoxicity during fluorophore excitation, the ability to gain complementary data such as membrane potential, membrane integrity, and DNA degradation may be invaluable in advancing the drug discovery pipeline. Alternatively, brightfield imaging of microfluidic channels/chambers offers a simple, direct, and dye-free measurement of cell viability (Sun et al., 2014) through bacterial cell counts and may provide insight into the antibacterial mechanism of action (Uddin et al., 2017); thus, brightfield imaging is a preferred metric for cell viability during microfluidic platform optimization. Regardless, brightfield imaging still presents analytical challenges, where the imaging of only a cross-section of a three-dimensional channel and the inhomogeneity of cells throughout microfluidic channels/chambers can mislead the accurate interpretation of bacterial growth (Golchin et al., 2012).

Herein, a microfluidics-based bioactivity assay is compared with that of a traditional bulk 96-well plate in order to assess its potential to elevate PepSAVI-MS AMP identification and biological characterization. A natural product peptide library with previously reported antibacterial bioactivity (Parsley et al., 2019) was generated from the cyclotide-expressing botanical species *Viola inconspicua* and screened against *Escherichia coli* ATCC 25922. Bioactivity is determined through a polymethylmethacrylate (PMMA) microfluidic platform coupled with confocal brightfield microscopy and compared to that of polypropylene (PP) and polystyrene (PS) plate-based assays using both optical density and resazurin-based fluorescence measurements. Despite similarities in the activity profiles of PP and PS plate-based assays, *V. inconspicua* library bioactivity was highly variable among plate-based and microfluidic platforms. These results show that significant optimization must be pursued prior to the incorporation of microfluidic technologies into established antimicrobial susceptibility pipelines.

## Methods

### Plant Material

*Viola inconspicua* seeds (Mountain Gardens, Burnsville, NC) were grown in a laboratory greenhouse (14/10 light/dark, 17.5–20.3 °C) to mature rosettes. *Viola inconspicua* greenhouse specimens have been submitted to the UNC Herbarium under accession numbers NCU00303107 and NCU00303108 and can be viewed on the SERNEC (Southeast Regional Network of Expertise and Collections) website (http://sernecportal.org/portal/index.php).

### Peptide Library

**Figure 1:**
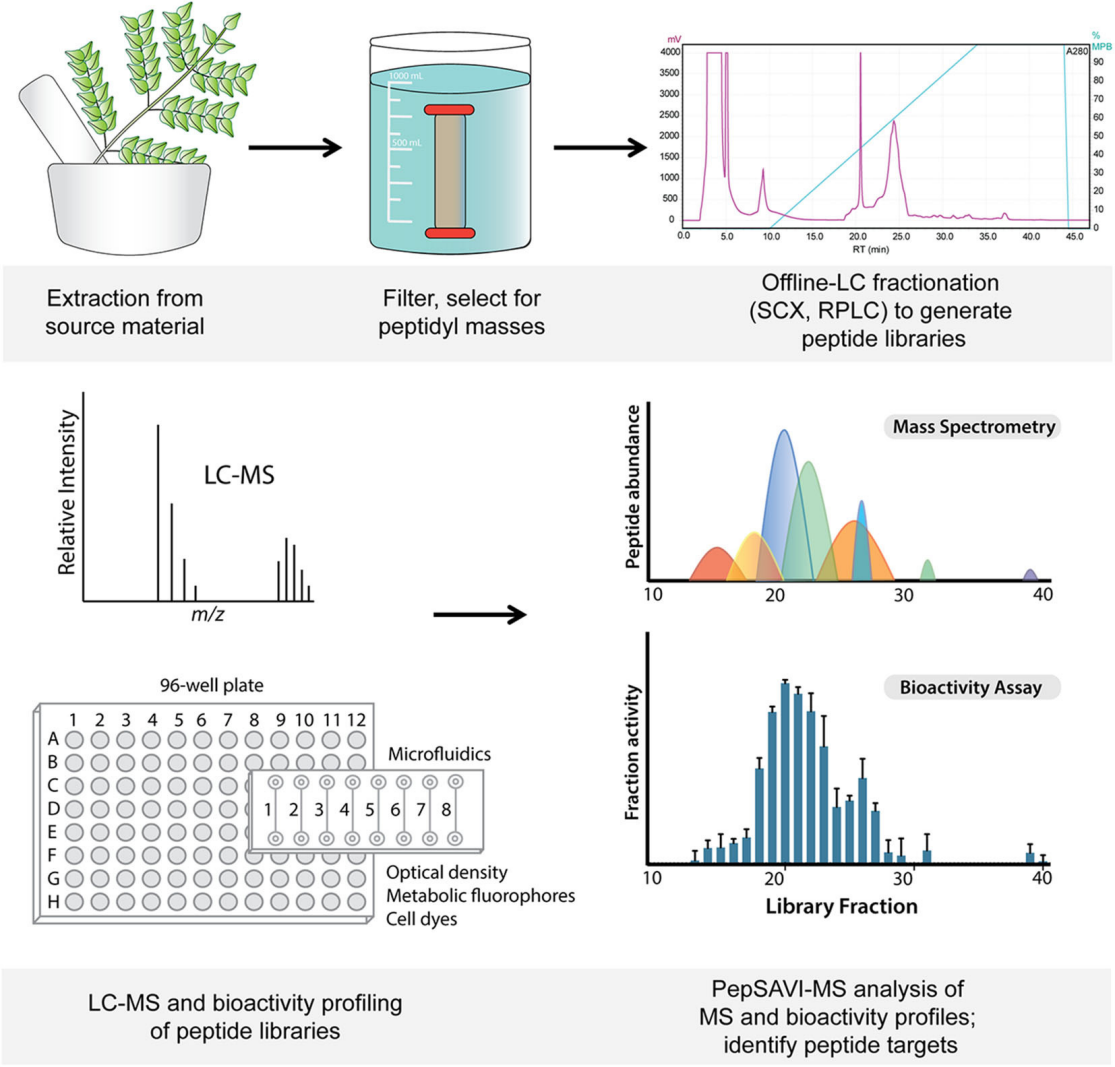
Workflow from starting material extraction through filtering and fractionation steps, generating a peptide library (top). Libraries are screened via LC-MS and for bioactivity (bottom), and subsequent PepSAVI-MS statistical analysis guides the identification of putative bioactive peptide species.

Plant material was extracted and peptide library prepared as previously described (Kirkpatrick et al., 2017; Parsley et al., 2019) (Figure 1). Briefly, *V. inconspicua* leaf tissue was harvested, ground under liquid nitrogen, and extracted into 10% acetic acid (1:3 w/v, 4 h, 4 °C, with stirring). Extract was sterile-filtered (0.22 μm, Corning), filtered to remove high molecular weight components (30 kDa MWCO, Millipore), and dialyzed into 5 mM ammonium formate, 20% acetonitrile, pH 2.7 to remove molecules < 1 kDa (0.1–1 kDa MWCO, SpectrumLabs). Remaining small molecule components were removed by injecting the extract onto strong cation exchange (PolySulfoethyl A column, 100 mm × 4.6 mm, 3 μm particles, PolyLC) using a linear salt gradient (5 mM ammonium formate, 20% acetonitrile, pH 2.7 to 500 mM ammonium formate, 20% acetonitrile, pH 3.0) and a flow rate of 0.5 mL/min. All late-eluting peptide-containing fractions, as later retention times are characteristic of cyclotides, were collected and combined, desalted with a C_18_ solid-phase extraction (100 mg, Waters Sep-Pak), dried down in a vacuum concentrator, and resuspended in water. A *V. inconspicua* reversed-phase peptide library was prepared by injecting this sample onto a reversed-phase column (Phenomenex Jupiter C_18_ column, 300 Å, 5 μm, 150 mm × 4.6 mm) where mobile phase A (MPA) consisted of 95/5 water/acetonitrile with 0.1% TFA; mobile phase B (MPB) consisted of acetonitrile with 0.1% TFA, using the following gradient: 0% MPB from 0 to 10 m, 0 to 20% MPB from 10 to 13 m, 20 to 40% MPB from 13 to 33 m, 40 to 100% from 33 to 43 m, and hold at 100% B from 43 to 47 m, a flow rate of 0.5 mL/min, and collecting 1 min fractions. *Viola inconspicua* peptide reversed-phase library fractions were dried down via vacuum centrifugation and resuspended in acidified LC-MS grade water.

### LC-MS/MS Analysis

*Viola inconspicua* library fractions 34–39 (~1 μg) were injected onto a nano-LC-ESI-MS/MS consisting of a NanoAcquity LC (Waters, Milford, MA) coupled to a TripleTOF5600 MS (AB Sciex, Framingham, MA). Front-end UPLC separation of peptides was accomplished via a Symmetry C18 trap column (100 Å, 5 μm, 180 μm × 20 mm, Waters) and an HSS T3C18 column (100 Å, 1.8 μm, 75 μm × 250 mm, Waters), with a flow rate of 0.3 μL/min and a 30 min linear ramp of 5–50% B (mobile phase A, 1% formic acid in water; mobile phase B, 1% formic acid in acetonitrile). The TripleTOF5600 MS was operated in positive-ion, high-sensitivity mode, and an IDA method with a cycle time of 2 s, the MS survey spectrum using a mass range of 350–1,600 Da in 250 ms, and the first 20 *m/z* from low to high *m/z* selected for MS/MS, acquiring each MS/MS over ~87 ms. Deisotoped peak lists for each library fraction were generated using Progenesis QI for Proteomics software (Nonlinear Dynamics, v.2.0) with a retention time filter of 14–45 min and a maximum charge of +10. “Peptide ion data” was exported from Progenesis and used to inform the relative abundances of *m/z* across *Viola inconspicua* library fractions.

### Bacterial Cultures

*Escherichia coli* ATCC 25922 frozen glycerol stocks were streaked onto Mueller-Hinton broth (MHB, BD difco) agar plates and grown overnight at 37 °C. A colony from each plate was used to inoculate 5 mL of MHB in a culture tube, grown 8–16 h at 37 °C, 250 rpm, and subsequently used to inoculate plate-based assay and microfluidic samples as described below.

### Plate-Based Assay

PP and PS 96-well plates were used to assess *V. inconspicua* library bioactivity in triplicate. Bacteria were adapted to liquid growth as described above, diluted back to OD_600_ = 0.25 in MHB, and grown for 1 h prior to the experiment to regain active growth. Each sample assayed consisted of 10 μL bacterial culture (diluted for a final concentration of OD_600_ = 0.1 in well), 20 μL 1X MHB, 10 μL 2X MHB, and 10 μL *V. inconspicua* library fraction. *V. inconspicua* library fractions 34–39 were assayed and positive and negative controls were prepared by replacing library fraction with ampicillin and water, respectively. Plate assays were incubated at 37° C for 4 h, shaking at 250 rpm. At *t* = 4 h, OD_600_ reads were acquired on a SpectraMax M5 (Molecular Devices). For fluorescence analysis, 1 μL resazurin sodium salt (Sigma) was added to each well for a final concentration of 1 mM and the plates were incubated at 37° C, 250 rpm. After 1 h or 1.5 h (*t* = 5 h or 5.5 h total assay time) for PS and PP plates, respectively, fluorescence of each well was measured (SpectraMax M5), with excitation at 544 nm and emission at 590 nm.

### Microfluidics

An 8-channel PMMA microfluidic chip (Microfluidic-Chipshop, Germany) with channels 100 μm × 100 μm × 18 mm and PP Luer fluidic interface was used for the microfluidic assay in singlet. Each channel was flushed with 100 μL 2.5 M NaOH, 100 μL sterile water, and equilibrated with 100 μL MHB prior to inoculation. After initial adaptation to liquid growth as described above, bacterial cultures were diluted to OD_600_ = 0.5 in MHB. Approximately 40 μL of each sample was loaded into a channel via pipette tip (until excess flowed through the outlet), consisting of the same proportions of bacterial culture (diluted to a final in-chip concentration of OD_600_ = 0.1), 1X MHB, 2X MHB, and *V. inconspicua* library fraction. *V. inconspicua* library fractions 34–39 were assayed and positive and negative controls were prepared by replacing library fraction with ampicillin (100 μg/mL final concentration) and water, respectively. The microfluidic chip was incubated at room temperature for 1 h prior to bacterial growth assessment via confocal brightfield imaging.

### Microscopy

Brightfield contrast and fluorescence images were acquired on a Zeiss LSM710 confocal laser-scanning microscope, with PlanApo 40X and 10X 0.45NA objective lenses, respectively. Brightfield and fluorescence images were collected using 561 nm and 405 nm lasers, respectively, on Zeiss ZEN software (Car Zeiss, INC. NY, USA). Bacterial cell viability was assessed via manual counting of bacterial cells in brightfield images.

### Cell Counts

Differences in cell counts (Δcell) were calculated by subtracting initial cell counts from final cell counts for each replicate, averaged, and standard deviation calculated. Bioactivity calculations were performed using Δcell counts for each channel, each replicate, in the following equation:

(1) $$\begin{array}{l} \%\;Activity\\ =\left[1-\left(\frac{V.inconspica\;fraction\;\Delta cell-positive\;control\;\Delta cell}{negative\;control\;\Delta cell-positive\;control\;\Delta cell}\right)\right]\\ \;*100 \end{array}$$

## Results and Discussion

### Cyclotide Library

**Figure 2:**
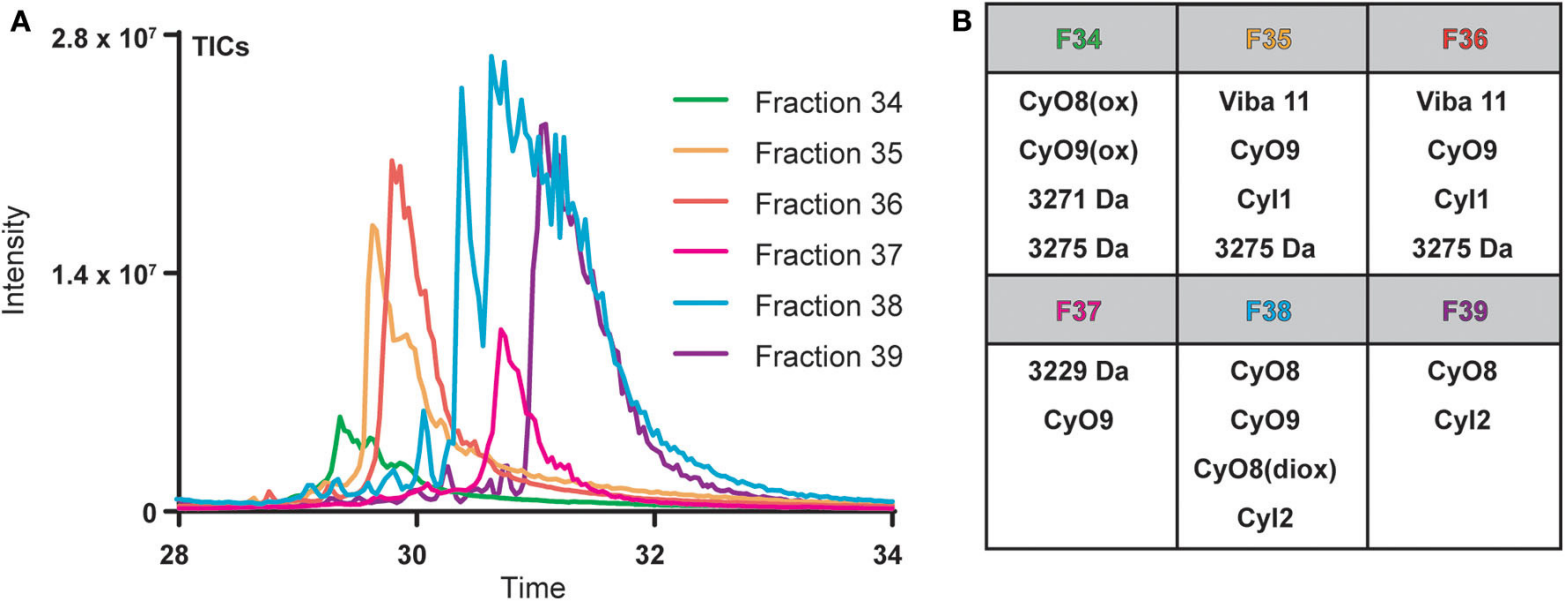
Overlaid total ions chromatograms (TICs) of *V. inconspicua* peptide library fractions 34–39 **(A)** and cyclotide constituent analysis of each fraction **(B)**. Oxidized and doubly oxidized species are denoted by (ox) and (diox), respectively.

Cyclotide-containing peptide libraries consistently demonstrate potent antibacterial activity in 96-well plate assay format (Parsley et al., 2019), and were thus utilized for comparison analyses of plate-based and microfluidics activity assays. *V. inconspicua* library fractions 34–39 were analyzed for peptidyl constituents via LC-MS and chosen for the high intensity UV traces and late retention time characteristic of cyclotide species. Mass spectral (MS1) analysis revealed that all library fractions contain masses corresponding to cyclotides or putative cyclotide species previously identified in *V. inconspicua* (Figure 2) (Parsley et al., 2019). Highly abundant masses present in the *V. inconspicua* library fractions are consistent with the masses of the fully characterized cyclotides cyO8 (fractions 38–39), cyO9 (fractions 35–38), viba 11 (fractions 35–36), cyI1 (fractions 35–36), cyI2 (fractions 38–39), and cyO8/cyO9 oxidized variants (fractions 34/38). Additional high abundance masses 3271 Da (fraction 34), 3275 Da (fraction 34-36), and 3229 Da (fraction 37) were identified as putative cyclotide species based on a mass shift analysis (Parsley et al., 2019), however, these sequences have not yet been characterized. The presence of different cyclotide species throughout the fraction subset, with at least one cyclotide mass in every fraction, and the propensity of cyclotides to exhibit antimicrobial activities, supports the use of this fraction library for assay comparisons.

### Bioactivity of 96-Well Plate Assays

**Figure 3:**
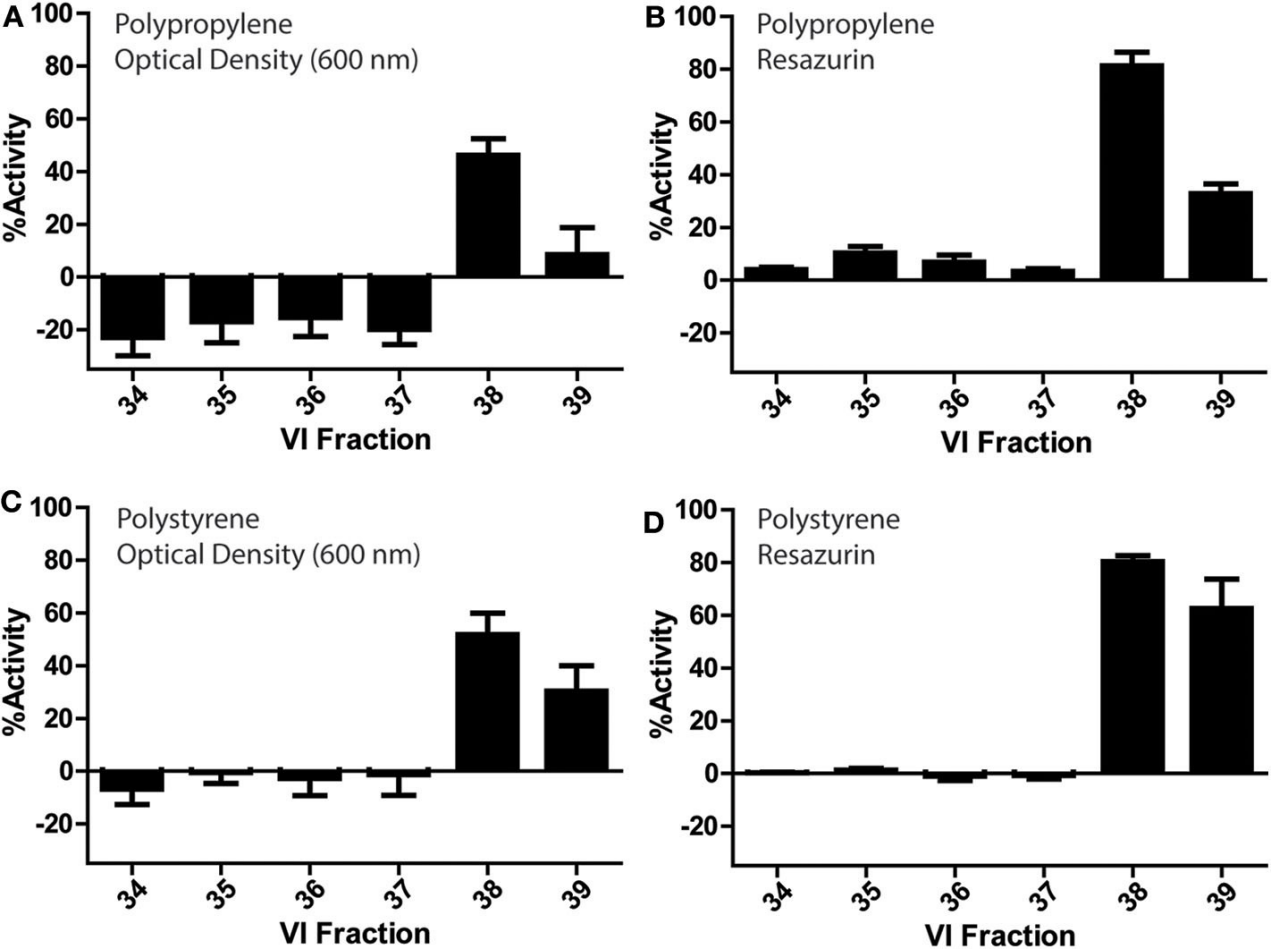
Bioactivity profiles of a *V. inconspicua* library against *E. coli* ATCC 25922 in a 96-well plate format and cell viability assessment with optical density in polypropylene **(A)**, fluorescence in polypropylene **(B)**, optical density in polystyrene **(C)**, and fluorescence in polystyrene **(D)**. Assays performed in triplicate, error bars represent standard deviation.

*V. inconspicua* library fractions 34–39 were assessed for bioactivity in conventional PP and PS 96-well plates (Figure 3). Optical density and fluorescence measurements conferred significant bioactivity in the *V. inconspicua* library fractions 38 and 39. However, activity was dependent on the measurement of cell viability (absorbance vs. fluorescence) and the plate material (PP vs. PS). Among the active fractions in the PP and PS plate assays, optical density reads averaged 60 ± 5 and 37 ± 16% less bioactivity than fluorescent reads for fractions 38 and 39, respectively; accordingly, fluorescent resazurin/resorufin measurements can achieve > 6-fold higher signal to background (Kim and Jang, 2018) in comparison to O.D. measurements (Vukomanovic and Torrents, 2019). Polypropylene and PS optical density-based activity measurements of fraction 38 were within error of each other, at 46 ± 6 and 51 ± 8% activity, respectively. Likewise, fluorescence measurements of fraction 38 for both plate materials were within error with 81 ± 5 and 80 ± 2% for PP and PS. However, significant differences were seen in the bioactivities of PP O.D. and fluorescence measurements of fraction 39, PS O.D. and fluorescence measurements of fraction 39, and the non/less active fractions 34–37 in the PP plate. Significant growth promotion was seen only in the PP O.D. measurements of fractions 34–37. Generally, both PP and PS O.D. reads exhibited higher standard deviations in activity measurements vs. fluorescence-based measurements, reflecting the inhomogeneity of bacterial cultures.

### Bioactivity in Microfluidic Assays

*V. inconspicua* library fraction activity was assessed with a simple 8 straight-channel microfluidic chip (Figure 1) to explore the merits of a microfluidic bioassay format. Whereas traditional “bulk” assays (e.g., 96-well plate) often require a minimum of 50 μL of sample mixture in each well (Sarker et al., 2007; Teh et al., 2017; Fowler et al., 2018) (10 μL extract, 40 μL of media and cells for each replicate) (Kirkpatrick et al., 2017; Parsley et al., 2018, 2019; Moyer et al., 2019), microfluidic devices utilize sample sizes in the nanoliter/picoliter range. However, connectors and interfaces between microfluidic channels/chambers and injection sites can generate significant dead volume, requiring excess sample to ensure complete filling of microfluidic compartments. Although <1 μL was theoretically needed to fill each microfluidic test channel, 25 μL was used during injection to completely fill the channel and Leur tip-fitted wells, only decreasing the required sample quantity by a factor of two when compared with a 96-well plate format. Additionally, microfluidic chips are made from plastic materials, e.g., PMMA or polydimethylsiloxane (PDMS), that are prone to degradation upon reuse and deformations during manufacturing. These imperfections were visible under a confocal microscope and contributed to varying refractive indices, resulting in poor image quality and reduced standardization.

**Figure 4:**
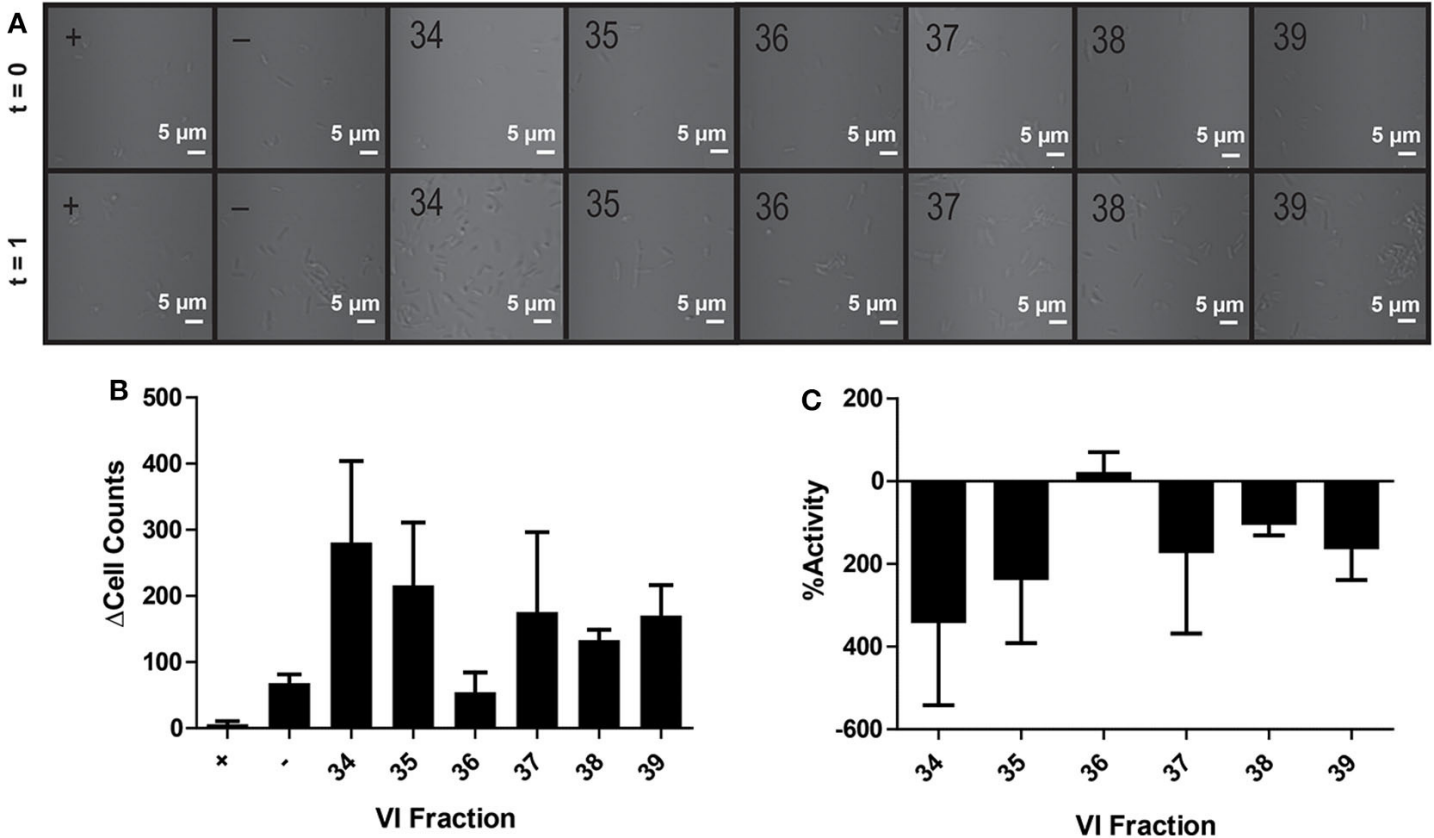
Activity of peptide fractions as measured with brightfield microscopy. Microfluidic channels are imaged at the initial assay time point and after 1 h of growth **(A)**. Differences in initial to final cell counts are calculated from brightfield images in positive control (+), negative control (–), and *V. inconspicua* library fraction replicates **(B)**. The percent bioactivities of *V. inconspicua* fractions are calculated from cell count differences **(C)**.

In contrast to the plate-based assays, no bioactivity was observed in the microfluidic chip—with growth promotion dominating in most channels (Figures 4A–C). Extreme growth variation among experimental replicates is seen by large standard deviations in bacterial growth across all channels; only positive control antibiotic-containing channels showed negligible deviation in cell count differences (final-initial cell counts) among experimental replicates, where averaging even negative control (water) fractions resulted in an average cell difference (Δcell counts) of 68 ± 23 cells (Figure 4B). Notably, despite care to homogenize master sample mixes prior to loading, samples within the channel were highly heterogeneous. Large variations in cell counts among focal planes and chamber latitudes and visible cell clumping at random intervals were likely the source of inaccurate or erratic cell counts throughout this experiment. While using Z-stacks could enable counting across focal planes, the imperfections in fabricated microfluidic chips prevented standardized Z-stacks across different channels. Poor reproducibility across replicates was likely a result of channel imperfections, varying refractive indexes, and the three-dimensional nature of the channel.

### Comparison of Plate-Based and Microfluidic Assays

Bioactivity differences in fractions 38/39 between plate-based and microfluidic assays were striking. The microfluidic chip used was comprised of PMMA with PP Leur connectors on the inlets/outlets of the device, while the 96-well assay plates were comprised of PP and PS. Polymethylmethacrylate is a biocompatible hydrophilic plastic, while PP/PS are inexpensive hydrophobic plastics used to produce the majority of assay plates. Thus, hydrophobic/amphipathic biomolecules (e.g., AMPs) are subject to extensive PP/PS surface binding via hydrophobic interactions (Jones et al., 2012; Stromstedt et al., 2014). Though PP materials bind polar molecules less than PS, the opacity of PP hinders the measurement of bacterial growth via optical density reads (Stromstedt et al., 2014). The surface properties of PMMA, including charge densities and reactive functional groups, are significantly impacted by the initial fabrication method and temperature, with potential impact on cell growth, homogeneity, and bioavailability of bioactive compounds (Becker and Locascio, 2002; Horinek et al., 2008). In any case, subsequent activity assays may benefit from passivation of assay plates/chips with inert biomolecules (e.g., polyethylene glycol, bovine serum albumin) prior to bacterial inoculation and bioactivity assessment in order to lessen the effect of surface interactions on experimental results. Undoubtedly, further comparisons of microfluidic and plate-based activity assays will be performed to probe the utility/feasibility of moving to a microscale assay format; such future assays may benefit from an estimation of assay sensitivity using standard AMPs with differing chemistries.

### Hydrophobicity

The *V. inconspicua* peptide library used in all analyses was generated via reversed-phase LC, where later eluting fractions (e.g., 38/39) contain hydrophobic cyclotide species. Major concerns regarding peptide non-specific binding to plastic surfaces (Chico et al., 2003; Goebel-Stengel et al., 2011) may explain a decrease in peptide concentration of hydrophobic peptides in hydrophobic activity assay plates. However, our experimental results counter this reasoning, as increasingly hydrophobic cyclotides are active in assays with hydrophobic material (PP/PS). Surface binding interactions are complicated by the propensity for cyclotides to oligomerize; for example, the cyclotide kalata B2 self-associates into tetramers and octamers in buffer where the 3-D hydrophobic face characteristic of cyclotides is “quenched,” (Rosengren et al., 2013) and this behavior can affect surface binding interactions (Ballet et al., 2010). It can therefore be surmised that peptide binding is not only determined by hydrophobic interactions; rather, a complex combination of hydrophobic interactions, peptide conformations and orientations, as well as media, can affect the susceptibility of peptide adsorption to various surfaces (Roach et al., 2005; Horinek et al., 2008).

### Oxygenation

Oxygenation plays an important role in bacterial growth, response to environmental cues, and antibiotic susceptibility (Gupta et al., 2016). Significantly different oxygen levels are experienced in microfluidic systems and plate-based assays. PMMA has low gas permeability (Ochs et al., 2014), with longer assays (>1 h) requiring an infusion of new media containing dissolved oxygen to support bacterial growth. Contrastingly, plate-based assays incorporate considerably more oxygen when shaking during incubation. The clinical relevance of bioactivity assay formats must be assessed: while assays are typically performed in ambient oxygen levels, decreased oxygen may be more representative of certain clinical environments (e.g., burns, abscesses, oral cavity) (Gupta et al., 2016). Although measured activity differences between microfluidic and plate-based assays are striking and could originate in part from the increased oxygenation with 96-well plates, producing the same activity profiles in different bioassay formats may be irrelevant as no assay format imitates true biological conditions. While there is no substitute for dynamic biological systems, *in vivo* systems are also flawed: animal models often do not correlate directly to human biology and cannot resolve the same mechanistic detail as *in vitro* assays (Petronilho et al., 2012). As such while challenges exist between high throughput bioactivity assays, they are still the first line of identification and characterization of novel antimicrobial compounds.

## Conclusion

Metrics of compound bioactivity include a host of assay platforms and reagents, where specialty assay designs and smart combinations of dyes and fluorophores allow us to peer beyond basic bioactivity assessment into the mechanisms of action and specific cellular effects of antimicrobial treatments. While microfluidic technologies are an emerging alternative to conventional bulk activity assays with the potential for high-throughput antimicrobial compound discovery and characterization (Liu et al., 2017), the integration of microfluidic devices into current drug discovery platforms requires careful parameter consideration and significant optimization. Polypropylene and PS plate-based assays largely confer similar bioactivity profiles for matched samples. However, dramatic differences in bioactivity for plate-based vs. microfluidic assays are observed; where the latter demonstrated no significant activity and growth promotion in all channels. Though the basis of these differences is unclear, major influences could be derived from (a) the plastic materials comprising each assay device and/or (b) the growth/assay conditions. Importantly, the biological relevance, the benefit of a specific assay type, and the merit of using multiple assay platforms for orthogonal compound identification is not clear, and may be complicated by AMP/pathogen identity. Microfluidic devices may offer an alternative activity assay form with the potential for increased experimental flexibility in design and output; however, significant optimization of a microfluidic platform is required before standardization and common laboratory use.

## Data Availability Statement

The datasets generated for this study can be found in the PRIDE repository, Project Name: Implementation of microfluidics for antimicrobial susceptibility assays: issues and optimization requirements, Project accession: PXD020857.

## Author Contributions

NP conceptualized experiments. NP and AS designed and performed the experiments, analyzed and interpreted the data, and wrote the original manuscript draft. All authors contributed to manuscript revision, read, and approved the submitted version.

## Conflict of Interest

The authors declare that the research was conducted in the absence of any commercial or financial relationships that could be construed as a potential conflict of interest.
